# Gonorrhoea and male bladder cancer in a prospective study

**DOI:** 10.1038/sj.bjc.6603510

**Published:** 2006-12-12

**Authors:** D S Michaud, E A Platz, E Giovannucci

**Affiliations:** 1 Department of Epidemiology, Harvard School of Public Health, Boston, MA, USA; 2 Channing Laboratory, Department of Medicine, Brigham and Women's Hospital and Harvard Medical School, Boston, MA, USA; 3 Department of Epidemiology, Johns Hopkins Bloomberg School of Public Health, Baltimore, MD, USA; 4 Brady Urological Institute, Johns Hopkins Medical Institutions, Baltimore, MD, USA; 5 Sidney Kimmel Comprehensive Cancer Center, Johns Hopkins Medical Institutions, Baltimore, MD, USA; 6 Department of Nutrition, Harvard School of Public Health, Boston, MA, USA

**Keywords:** gonorrhoea, infection, inflammation, bladder cancer, cohort study

## Abstract

In a prospective cohort study, a close to two-fold elevated risk of bladder cancer was found among men reporting a history of gonorrhoea (relative risk=1.92, 95% CI=1.10–3.33). Our finding warrants further examination of the role of gonorrhoea in bladder carcinogenesis.

Bladder cancer is the fourth most common cancer in men, and the sixth most commonly diagnosed cancer in the US (Ries *et al*, 2006). Schistosomiasis is a well-established risk factor for bladder cancer and explains high rates of bladder cancer in regions where schistosomiasis is endemic; in these regions, squamous cell carcinoma is the most common histologic subtype (Mostafa *et al*, 1999). To date, it remains unclear whether infection and/or inflammation play a role in bladder carcinogenesis in developed countries where transitional cell carcinoma is the most common histologic type.

Gonorrhoea is the second commonest sexually transmitted disease in the US (CDC, 2005). Infection by the bacterium *Neisseria gonorrhoeae* commonly presents as acute urethritis in men, whereas in women, it initiates in the uterine cervix and is often asymptomatic (Edwards and Apicella, 2004). Urethritis among men with gonorrhoea frequently recurs, even after initial treatment (Bowie, 1990), often causing recurring inflammation of the urethra. The inflammation caused by such infection may play a role in bladder carcinogenesis, and two case–control studies have reported increased bladder cancer risk associated with a history of gonorrhoea: in one, invasive transitional cell carcinoma showed a relative risk (RR) of 2.1 (95% CI=1.0–4.5), after adjusting for age, sex, and smoking (La Vecchia *et al*, 1991), and in the other, bladder cancer RR was 2.42 (95% CI=1.00–5.83), after adjusting for age and geographic location (91.5% of cases being invasive) (Mommsen and Sell, 1983). Because both studies assessed exposure after the diagnosis of bladder cancer, recall or selection bias cannot be ruled out as possible explanations for the observed findings.

We examined the possible role of gonorrhoeal infection in bladder cancer, while minimising possible bias in a prospective cohort study of men.

## MATERIALS AND METHODS

### Study population

The Health Professionals Follow-Up Study was initiated in 1986, when 51 529 predominantly white men aged 40–75 years and living in all 50 US states answered a detailed mailed questionnaire on medical history, diet and other characteristics. Deaths of cohort members are frequently reported by family members or by the postal service in response to questionnaire mailings. In addition, the National Death Index is searched biennially for non-respondents; this method has been shown to have a sensitivity of 98% (Rich-Edwards *et al*, 1994). Through 2002, the total follow-up rate for this cohort is greater than 96%.

At baseline, and biennially thereafter, participants provided information on their current smoking status, medical conditions, and other characteristics. The baseline questionnaire included detailed information on past smoking habits, time since quitting, and average cigarettes smoked per day by decade.

This study was approved by the Human Subjects Committee of the Harvard School of Public Health.

### History of gonorrhoea

On the 1992 questionnaire, participants were first asked whether they had ever had a diagnosis of gonorrhoea or syphilis, or neither. A total of 3212 men (8.7%) did not respond to this specific question. Information on urinary tract infections (UTI) was not obtained from participants in this cohort. Prevalence of syphilis was too low to examine association with bladder cancer in this study.

### Cancer ascertainment

We confirmed self-reported diagnosis of bladder cancer with medical records (87% of cases). When permission to obtain medical records was denied, we confirmed the self-reported cancer and date of diagnosis from a secondary source (e.g., death certificate or physician). If the primary cause of death reported by the National Death Index was a previously unreported bladder cancer, we contacted family members to obtain permission to retrieve medical records, or at least, to confirm the diagnosis. Based on pathology reports, over 95% of bladder cancer cases were transitional cell carcinomas. We used the TNM classification system to stage the tumours. In a subanalysis, noninvasive papillary carcinomas (Ta) were separated from invasive tumours (T1–T4). Carcinoma *in situ* tumours (Tis) were included in the overall analysis as these lesions have a high risk of progressing (Dorkin *et al*, 1997), but were excluded from the subanalysis (as they have invasive potential). Overall, 286 incident bladder cancer cases with complete reported information on gonorrhoea were available for the analysis.

### Statistical analysis

For this analysis, we included 37 012 cohort participants who responded to the 1992 questionnaire and were alive and free of cancer (except nonmelanoma skin cancer) in 1992. We computed person-time of follow-up for each participant from the return date of the 1992 questionnaire to the date of bladder cancer diagnosis, death from any cause or 31 January 2002, whichever came first. We categorised participants according to history of gonorrhoea and included a missing category. We used Cox proportional hazards models to adjust for potential confounding variables, including age (5-year categories), pack-years of smoking, current smoking status, race, and region of residence. Analyses were conducted using SAS release 9.1 (SAS Institute, Cary, NC, USA).

## RESULTS

Participants who reported a history of gonorrhoea were slightly younger, more likely to be ever smokers, and more likely to be African-American than those with no history of infection. Participants who responded to the 1992 questionnaire but did not answer the STD question were similar to men with no history of infections across baseline characteristics, including indicators of medical care utilisation, with the exception that they were slightly older (Supplementary Data).

Men with a history of gonorrhoea had a two-fold increase in bladder cancer risk compared to men without a history of gonorrhoea (Table 1). Controlling for smoking history, race, region of residence, and total fluid intake attenuated the association slightly (RR=1.92, 95% CI=1.10–3.33). In a secondary analysis, the association between gonorrhoea and bladder cancer was slightly stronger after excluding the first 2 years (1992–1994) of follow-up (multivariable RR=2.07, 95% CI=1.19–3.60).

We stratified cases by tumour invasiveness to further examine the possibility that detection bias is responsible for the observed association. The association was not apparent in men with superficial bladder cancer (Ta; Table 1) and was weak among those with less advanced disease (Ta and T1; RR=1.14, 95% CI=0.50–2.59). It was stronger in men with invasive (T1–T4; Table 1) and advanced cases only (T2–T4; RR=4.07, 95% CI=1.35–12.3). Further restricting the analysis to invasive TCC resulted in similar results (RR=2.38, 95% CI=1.00–5.63).

The association with gonorrhoea appeared stronger among ever smokers, although the interaction was not statistically significant (*p*-interaction=0.14). Compared to never smokers without a history of gonorrhoea, ever smokers without a history of gonorrhoea had an RR of 2.19 (95% CI=1.66–2.89), never smokers with a history of gonorrhoea had an RR of 1.45 (95% CI=0.35–6.04), and ever smokers with a history of gonorrhoea had an RR of 4.84 (95% CI=2.58–9.07). The association with gonorrhoea was similar among ever smokers with less than 25 pack-years of smoking (RR=1.91, 95% CI=0.87–4.18, yes *vs* no history of gonorrhoea) and among men with 25+ pack-years of smoking (RR=2.32, 95% CI=0.95–5.68, yes *vs* no history).

## DISCUSSION

In this prospective study, we observed a two-fold increase in bladder cancer risk among men with a history of gonorrhoea. The association was stronger for invasive and advanced bladder cancer and among current smokers.

Chronic or recurring urinary infections may increase bladder cancer risk through inflammation or urinary stasis. Urinary tract infections have been associated with bladder cancer risk in many case–control studies (Howe *et al*, 1980; Kantor *et al*, 1984; Claude *et al*, 1986; Piper *et al*, 1986; Gonzalez *et al*, 1991; La Vecchia *et al*, 1991). In one study, increased risk was observed among individuals who reported their first UTI 15 or more years before diagnosis (OR=2.3, 95% CI=1.0–5.2) (La Vecchia *et al*, 1991). In another case–control study, associations with UTI were strongest for advanced cancers (OR=4.6, 95% CI=2.4–8.9) (Kantor *et al*, 1984).

Lower urinary tract symptoms include incomplete bladder emptying, frequency, intermittency, urgency, and hesitancy, and we recently reported a positive association between history of gonorrhoea and such symptoms in this cohort (RR=1.63, 95% CI=1.14–2.33) (Sutcliffe *et al*, 2005). Based on these findings, gonorrhoeal infections, generally acquired relatively early in adulthood, may have a long-lasting impact on bladder function. It is plausible therefore that the inflammation producing these urinary symptoms, or increased urinary stasis from incomplete bladder emptying, or a combination of these could be involved in bladder carcinogenesis.

Although the interaction with smoking was not statistically significant in the current study, we observed a strong association with gonorrhoea and bladder cancer among ever smokers. The strength of the association among individuals with a high or a low pack-year smoking history was similar, suggesting that residual confounding by number of pack-years was unlikely. Effect modification by smoking of the UTI and bladder cancer association has been reported in two studies (Kantor *et al*, 1984; La Vecchia *et al*, 1991). In one, when compared to never smokers with no history of UTI, ever smokers with a history of UTI had an RR of 10.3 (95% CI=5.3–20.1), never smokers with a history of UTI had an RR of 3.2 (95% CI=1.6–6.3), and ever smokers with no history of UTI had an RR of 2.4 (95% CI=1.6–3.6) (La Vecchia *et al*, 1991).

The strengths of this study include its prospective design, which precludes recall bias, a large number of bladder cancer cases, and high follow-up rates. In addition, we conducted secondary analyses to address potential biases that may have occurred. We explored the possibility that a person with a history of gonorrhoea might be more likely to get a medical work-up that would lead to the detection of bladder cancer. If detection bias was present, we would expect less advanced or noninvasive tumours to be more commonly reported among those with a history of gonorrhoea. However, we observed stronger associations between gonorrhoea and bladder cancer risk among those who had advanced or invasive disease and no association among those with noninvasive disease. Furthermore, the association strengthened after removing the first 2 years of follow-up, suggesting that detection bias or reverse causation is unlikely to explain our findings.

As with other STDs, under reporting is probable, especially in face-to-face interviews where individuals may be uncomfortable reporting a positive history. Our data on gonorrhoea was obtained from mailed questionnaires and fewer than 9% of participants did not answer that question; we do not know why these men did not respond, but it is unlikely that nonrespondents were all positive for gonorrhoea. In this cohort, 2.9% of men reported a history of gonorrhoea, which is slightly higher than the prevalence rate reported in a case–control study on STD and prostate cancer among white men (2.5%) (Hayes *et al*, 2000). If all nonrespondents were positive, the prevalence of gonorrhoea would be 11% in this educated population of health professionals, which is unlikely. Furthermore, nonrespondents were similar to men with no history of gonorrhoea with respect to behavioural characteristics. Therefore, although we cannot rule out the possibility that some nonrespondents had gonorrhoea, it is unlikely to be a large proportion, and results would only be biased if the relationship between gonorrhoea and bladder cancer was different in responders and nonresponders.

In this cohort study of men, a history of gonorrhoea was associated with a statistically significant increase in bladder cancer risk (*P*-value=0.02). The 2.4-fold increase in risk observed for invasive disease and the lack of association among men with superficial disease suggest that detection bias is unlikely to explain our observation. Our findings from a prospective study confirm those of two previous case–control studies and warrant further study of the role of gonorrhoea and inflammation in bladder carcinogenesis.

## Figures and Tables

**Table 1:** Relative risks of bladder cancer according to history of gonorrhoea (overall and stratified by stage at initial diagnosis) in the Health Professionals Follow-up Study, 1992–2002

**History of gonorrhoea**	**No. of cases**	**Person-years**	**RR^a^ (95% CI)**	**MV RR^b^ (95% CI)**
No	272	307 159	1.0	1.0
Yes	14	9239	2.07 (1.19–3.57)	1.92 (1.10–3.33)^*^
				
*Among noninvasive (Stage 0a) tumours* c				
No	130	306 033	1.0	1.0
Yes	3	9166	0.89 (0.28–2.84)	0.87 (0.27–2.77)
				
*Among invasive (Stage 1+) tumours*				
No	99	305 748	1.0	1.0
Yes	7	9182	2.82 (1.28–6.19)	2.42 (1.09–5.38)^†^

a Age-adjusted relative risks; 95% CI=95% confidence intervals.

b Multivariable relative risks (MV RR) from proportional hazard models adjusting for age, pack-years of smoking, current smoking status, region, total fluid intake, and race.

c Noninvasive *in situ* (Stage 0is) tumours were excluded as these are typically more aggressive than the noninvasive papillary tumours. Cases with missing stage were excluded (*n*=57).

* *P*-value=0.02; ^†^*P*-value=0.03.

## References

[bib1] Bowie WR (1990) Approach to men with urethritis and urologic complications of sexually transmitted diseases. Med Clin N Am 74: 1543–1557 2246952 10.1016/s0025-7125(16)30494-1

[bib2] CDC (2005) Sexually Transmitted Disease Surveillance 2004. Atlanta: Centers for Disease Control and Prevention

[bib3] Claude J, Kunze E, Frentzel-Beyme R, Paczkowski K, Schneider J, Schubert H (1986) Life-style and occupational risk factors in cancer of the lower urinary tract. Am J Epidemiol 124: 578–589 3752052 10.1093/oxfordjournals.aje.a114430

[bib4] Dorkin TJ, Robson CN, Neal DE (1997) The molecular pathology of urological malignacies. J Pathol 183: 380–387 9496253 10.1002/(SICI)1096-9896(199712)183:4<380::AID-PATH959>3.0.CO;2-7

[bib5] Edwards JL, Apicella MA (2004) The molecular mechanisms used by Neisseria gonorrhoeae to initiate infection differ between men and women. Clin Microbiol Rev 17: 965–981, table of contents 15489357 10.1128/CMR.17.4.965-981.2004 PMC523569

[bib6] Gonzalez CA, Errezola M, Izarzugaza I, Lopez-Abente G, Escolar A, Nebot M, Riboli E (1991) Urinary infection, renal lithiasis and bladder cancer in Spain. Eur J Cancer 27: 498–500 1827729 10.1016/0277-5379(91)90395-t

[bib7] Hayes RB, Pottern LM, Strickler H, Rabkin C, Pope V, Swanson GM, Greenberg RS, Schoenberg JB, Liff J, Schwartz AG, Hoover RN, Fraumeni Jr JF (2000) Sexual behaviour, STDs and risks for prostate cancer. Br J Cancer 82: 718–725 10682688 10.1054/bjoc.1999.0986 PMC2363322

[bib8] Howe G, Burch J, Miller A, Cook G, Esteve J, Morrison B, Gordon P, Chambers L, Fodor G, Winsor G (1980) Tobacco use, occupation, coffee, various nutrients, and bladder cancer. J Natl Cancer Inst 64: 701–713 6928984

[bib9] Kantor AF, Hartge P, Hoover RN, Narayana AS, Sullivan JW, Fraumeni Jr JF (1984) Urinary tract infection and risk of bladder cancer. Am J Epidemiol 119: 510–515 6711540 10.1093/oxfordjournals.aje.a113768

[bib10] La Vecchia C, Negri E, D'Avanzo B, Savoldelli R, Franceschi S (1991) Genital and urinary tract diseases and bladder cancer. Cancer Res 51: 629–631 1985779

[bib11] Mommsen S, Sell A (1983) Prostatic hypertrophy and venereal disease as possible risk factors in the development of bladder cancer. Urol Res 11: 49–52 6191422 10.1007/BF00256946

[bib12] Mostafa MH, Sheweita SA, O'Connor PJ (1999) Relationship between schistosomiasis and bladder cancer. Clin Microbiol Rev 12: 97–111 9880476 10.1128/cmr.12.1.97 PMC88908

[bib13] Piper JM, Matanoski GM, Tonascia J (1986) Bladder cancer in young women. Am J Epidemiol 123: 1033–1042 3706274 10.1093/oxfordjournals.aje.a114331

[bib14] Rich-Edwards JW, Corsano KA, Stampfer MJ (1994) Test of the National Death Index and Equifax Nationwide Death Search. Am J Epidemiol 140: 1016–1019 7985649 10.1093/oxfordjournals.aje.a117191

[bib15] Ries L, Eisner M, Kosary C, Hankey B, Miller B, Clegg L, Mariotto A, Feuer E, Edwards BE (2006) SEER Cancer Statistics Review, 1975–2003. Bethesda, MD: National Cancer Institute

[bib16] Sutcliffe S, Giovannucci E, De Marzo AM, Willett WC, Platz EA (2005) Sexually transmitted infections, prostatitis, ejaculation frequency, and the odds of lower urinary tract symptoms. Am J Epidemiol 162: 898–906 16177142 10.1093/aje/kwi299

