# Supplementary material for: Gonorrhoea and male bladder cancer in a prospective study
Source: Br J Cancer. 2006 Dec 12;96(1):169–71. doi: 10.1038/sj.bjc.6603510 (PMC2360207; doi:10.1038/sj.bjc.6603510)
Supplement: Supplementary Data [file 6603510x1.doc]

**Supplemental Data**

Age-standardized characteristics of the Health Professionals Follow-up Study by history of gonorrhea, 1992.

History of Gonorrhea

No Yes Missing

### Cohort numbers 32,810 990 3212

**Age** (mean) 59.4 57.4 61.9

**Ancestry (%)**

South European 23 22 26

Scandinavian 10 9 10

Other Caucasian 58 55 54

African-American 1 7 1

Asian-American 2 1 2

Other/unknown 6 6 8

**Region**

West (%) 23 28 20

Midwest (%) 28 19 29

South (%) 27 34 26

Northeast (%) 23 19 26

**Smoking history (%)**

Never 46.2 33.8 47.0

<10 pack-years 9.6 10.6 10.3

10-24 pack-years 18.1 22.1 17.8

25-44 pack-years 13.3 17.0 11.6

45+ pack-years 7.6 11.3 6.8

Current smoker 6.7 9.0 6.2

**Medical history and exams**^*^ **(%)**

Physical exam, routine 62.0 59.5 61.5

Physical exam, symptomatic 17.0 18.9 17.0

Rectal exam, routine 60.0 61.3 59.2

Rectal exam, symptomatic 7.0 6.9 7.3

Vasectomy 22.1 23.6 21.2

History of syphilis 0 2 0

**Medication use (current, %)**

Multivitamins 41.6 45.3 40.6

Aspirin use 35.3 35.5 34.1

NSAID use 10.2 12.1 10.2

^*^Questions on physical and rectal exams were asked in 1992 and refer to the previous 2 year-period (i.e., between 1990 and 1992).
